# Integrating bioinformatic resources to identify characteristics of rheumatoid arthritis-related usual interstitial pneumonia

**DOI:** 10.1186/s12864-023-09548-2

**Published:** 2023-08-10

**Authors:** Yulu Qiu, Chang Liu, Yumeng Shi, Nannan Hao, Wenfeng Tan, Fang Wang

**Affiliations:** 1 Department of Rheumatology, The First Affiliated Hospital of Nanjing Medical University, Nanjing, 210029 China; 2 Department of Cardiology, The First Affiliated Hospital of Nanjing Medical University, Nanjing, Jiangsu China

**Keywords:** Bioinformatics analyses, Rheumatoid arthritis, Usual interstitial pneumonia, Immune pathological mechanism, Idiopathic pulmonary fibrosis

## Abstract

**Background:**

Rheumatoid arthritis (RA) is often accompanied by a common extra-articular manifestation known as RA-related usual interstitial pneumonia (RA-UIP), which is associated with a poor prognosis. However, the mechanism remains unclear. To identify potential mechanisms, we conducted bioinformatics analysis based on high-throughput sequencing of the Gene Expression Omnibus (GEO) database.

**Results:**

Weighted gene co-expression network analysis (WGCNA) analysis identified 2 RA-positive related modules and 4 idiopathic pulmonary fibrosis (IPF)-positive related modules. A total of 553 overlapped differentially expressed genes (DEG) were obtained, of which 144 in the above modules were further analyzed. The biological process of “oxidative phosphorylation” was found to be the most relevant with both RA and IPF. Additionally, 498 up-regulated genes in lung tissues of RA-UIP were screened out and enriched by 7 clusters, of which 3 were closely related to immune regulation. The analysis of immune infiltration showed a characteristic distribution of peripheral immune cells in RA-UIP, compared with IPF-UIP in lung tissues.

**Conclusions:**

These results describe the complex molecular and functional landscape of RA-UIP, which will help illustrate the molecular pathological mechanism of RA-UIP and identify new biomarkers and therapeutic targets for RA-UIP in the future.

**Supplementary Information:**

The online version contains supplementary material available at 10.1186/s12864-023-09548-2.

## Background

Rheumatoid arthritis (RA) is a prevalent systemic autoimmune disease characterized by synovial hyperplasia, persistent joint inflammation, and extra-articular manifestations. Among these manifestations, interstitial lung disease (ILD) is one of the most important extra-articular features, occurring in 19–67% of RA patients according to radiological assessments [1–3]. Moreover, ILD is the second leading cause of mortality in RA patients, following cardiovascular diseases.

Numerous studies have demonstrated that in RA-associated interstitial lung disease (RA-ILD), the typical radiological and histological pattern is usual interstitial pneumonia (UIP), which is associated with increased mortality compared to other patterns, such as non-specific interstitial pneumonia (NSIP) [4–6]. However, the precise pathogenesis of RA-UIP remains elusive, and effective preventive or curative measures for RA-UIP are currently lacking.

Idiopathic pulmonary fibrosis (IPF), a pathological UIP, is the most common type of pulmonary fibrosis and characterized by chronic progressive lung fibrosis. It is noteworthy that RA-UIP shares several similarities with IPF, including age, gender, environmental risk factors, imaging features of progressive lung fibrosis, and poor survival [4, 7–10]. The anti-citrullinated protein antibody (ACPA), a highly specific autoantibody found in RA patients, has been detected in the lungs of 46% of IPF patients [11, 12] and 44% of RA-ILD patients. Furthermore, the single nucleotide polymorphism (SNP) rs3570595016 in the promoter of MUC5B (mucin 5B, oligomeric gel-forming) has been identified as the strongest genetic risk factor for IPF. Interestingly, a recent study found that MUC5B promoter variant was robustly associated with an increased risk of RA-ILD and more specifically associated with UIP pattern on imaging [13]. These findings suggest that RA-UIP and IPF may share common cellular and molecular mechanisms.

The advancing field of omics is expected to identify the "molecular endotype", which could offer new insights into the relationship between the RA-UIP and IPF. Omics approaches can uncover shared or distinct biological pathways and processes that lead to the UIP fibrosis in these two disorders. In this study, we employed bioinformatics methods to investigate the characteristics of RA-UIP by analyzing gene expression profiles from three GEO datasets (GSE93272, GSE93606 and GSE199152). First of all, we identified common characteristics between RA and IPF through weighted gene co-expression network analysis (WGCNA) of whole blood samples (GSE93272, GSE93606), which were subsequently validated in pulmonary tissues of RA-UIP and IPF-UIP (GSE199152) and the collagen-induced arthritis (CIA) mice. Notably, Kyoto Encyclopedia of Genes and Genomes (KEGG) analyses and immune cell infiltration pattern mining in pulmonary tissues suggest that T-cell subsets and M1 macrophages may play an important role in the development of RA-UIP.

## Results

### GEO information

**Fig. 1:**
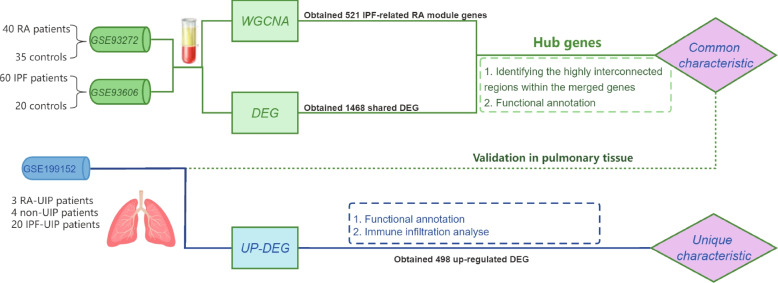
Flowchart of the work. Flowchart of the overall strategy of bioinformatics analyses

According to the established criteria mentioned in the methods section, three GEO datasets (GSE93272, GSE93606 and GSE199152) were selected. The information of the 3 datasets was summarized in Table 1, including GSE number, platform, sample, and types of RNA source. The GSE93272 (40 RA patients and 35 controls) and GSE93606 (60 IPF patients and 20 controls) were selected for the WGCNA analysis to explore the common characteristic of RA and IPF, and GSE199152 (3 RA-UIP, 20 IPF-UIP patients and 4 non-UIP controls) were used to validate the common characteristic in RA-UIP and IPF-UIP and explored the unique characteristic of RA-UIP. A flowchart of the overall strategy of bioinformatics analyses is shown in Fig. 1.

**Table 1:** Information for selected GEO datasets

ID	GSE number	Platform	Sample and disease	Source types
1	GSE93272	GPL570	40 RA patients and 35 controls	whole blood
2	GSE93606	GPL11532	60 IPF patients and 20 controls	whole blood
3	GSE199152	GPL16791	3 RA-UIP, 20 IPF-UIP patients and 4 non-UIP controls	pulmonary tissue

### The co-expression modules in RA and IPF

**Fig. 2:**
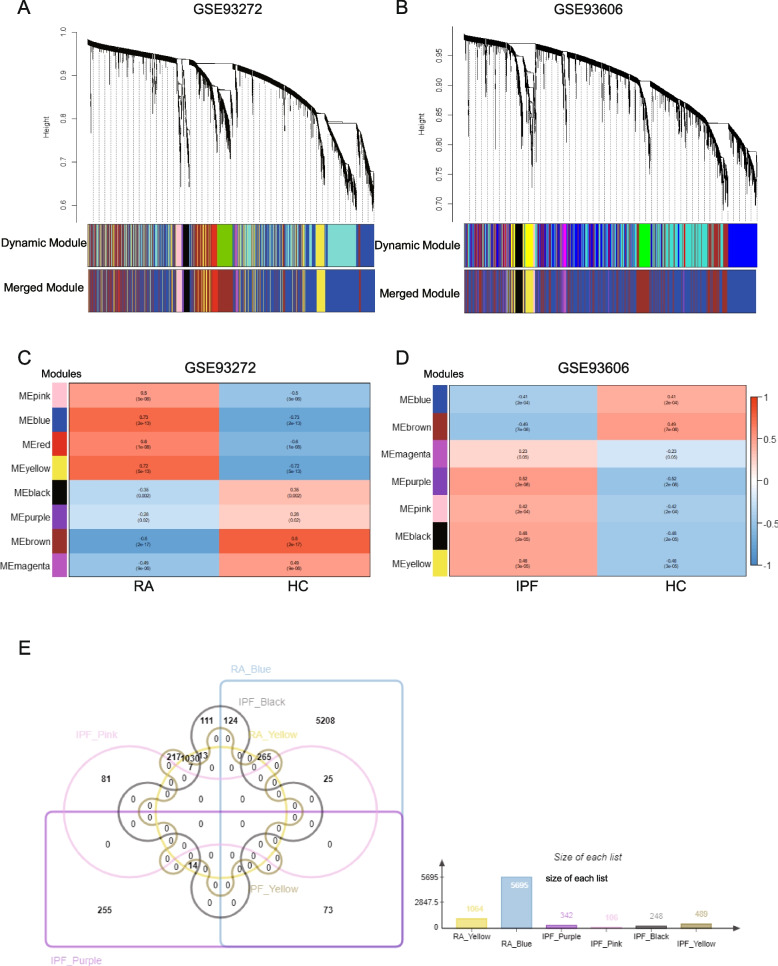
Weighted gene co-expression network analysis (WGCNA) of GSE93272 and GSE93606. **A** The cluster dendrogram of co-expression genes and modules in RA. **B** The cluster dendrogram of co-expression genes and modules in IPF. **C** Module–trait relationships in RA. Each row corresponds to a moduleEigengene, and the column corresponds to the patients or healthy controls. Each cell contains the corresponding correlation and *p*-value. **D** Module–trait relationships in IPF. Each row corresponds to a moduleEigengene, and the column corresponds to the patients or healthy controls. Each cell contains the corresponding correlation and *p*-value. **E** The shared genes between the modules of RA (yellow and blue) and the modules of IPF (purple, pink, black, and yellow) by overlapping them. RA, rheumatoid arthritis; IPF, idiopathic pulmonary fibrosis

The WGCNA resulted in the identification of eight and seven gene modules in GSE93272 and GSE93606, respectively. Each module was represented by a distinct color (Fig. 2A, B). The clustering dendrogram displayed the original and merged modules with similar expression patterns. The heatmap displayed the association between each module and the disease (Fig. 2C, D). In GSE93272, the blue (*r* = 0.73, *p* = 2e − 13) and yellow (*r* = 0.72, *p* = 5e − 13) modules were found to be positively correlated with RA-UIP, including 5695 and 1064 genes respectively (Fig. 2C, E). In GSE93606, the purple modules (*r* = 0.52, *p* = 2e − 6), pink modules (*r* = 0.42, *p* = 2e − 04), black module (*r* = 0.48, *p* = 2e − 05), yellow module (*r* = 0.46, *p* = 3e − 05) were positively correlated with IPF, including 342, 106, 248 and 489 genes respectively (Fig. 2D, E).

The common genes overlapped in RA-UIP-positive related modules and IPF-positive related modules were shown in Fig. 2E. The column chart shows the number of genes in each module. Detailed gene information was summarized in Supplementary Table S1, Additional file 2.

### Differential expression analysis of GSE93272 and GSE93606

**Fig. 3:**
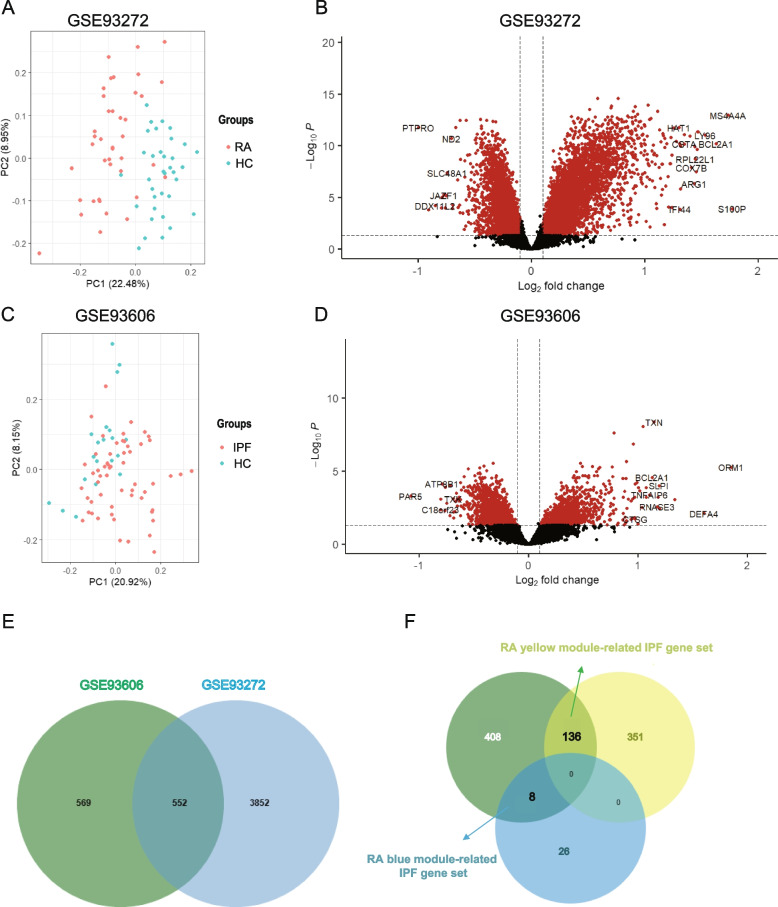
Differentially expression analysis of GSE93272 and GSE93606. **A** Principal component analysis shows that the RA patients and controls can be clearly distinguished. **B** The volcano plot illustrates DEGs between RA and control. **C** Principal component analysis shows that the IPF patients and controls can be clearly distinguished. **D** The volcano plot illustrates DEGs between IPF and control. **E** The shared DEG of GSE93272 and GSE93606. DEGs, differentially expressed genes. **F** Venn diagram shows the intersection of shared DEGs with RA blue module shared IPF genes and yellow module shared IPF genes

PCA demonstrated a different distribution pattern between the patients and controls, based on the expression of genes in all samples of the 2 datasets (Fig. 3A-C). Using the limma package in R software, 4404 upregulated DEGs between RA patients and controls in the GSE93272 dataset, and 1121 upregulated DEGs between IPF patients and control in the GSE93606 dataset were obtained under the criteria of *P* < 0.05 and log-FC > 0.1 (Fig. 3B-D). There are 553 overlapping upregulated DEGs shared by RA and IPF (Fig. 3E).

### The common characteristic of RA and IPF in whole blood

To screen out the shared genes with a significantly different expression, only the intersection part of RA-related IPF genes and overlapped DEGs were chosen for further analysis. Then, we obtained common 144 genes, and named them as two groups: the “RA yellow module related IPF gene set” (8 genes) and the “RA blue module related IPF gene set” (136 genes), respectively (Supplementary Table S2, Additional file 3).

**Fig. 4:**
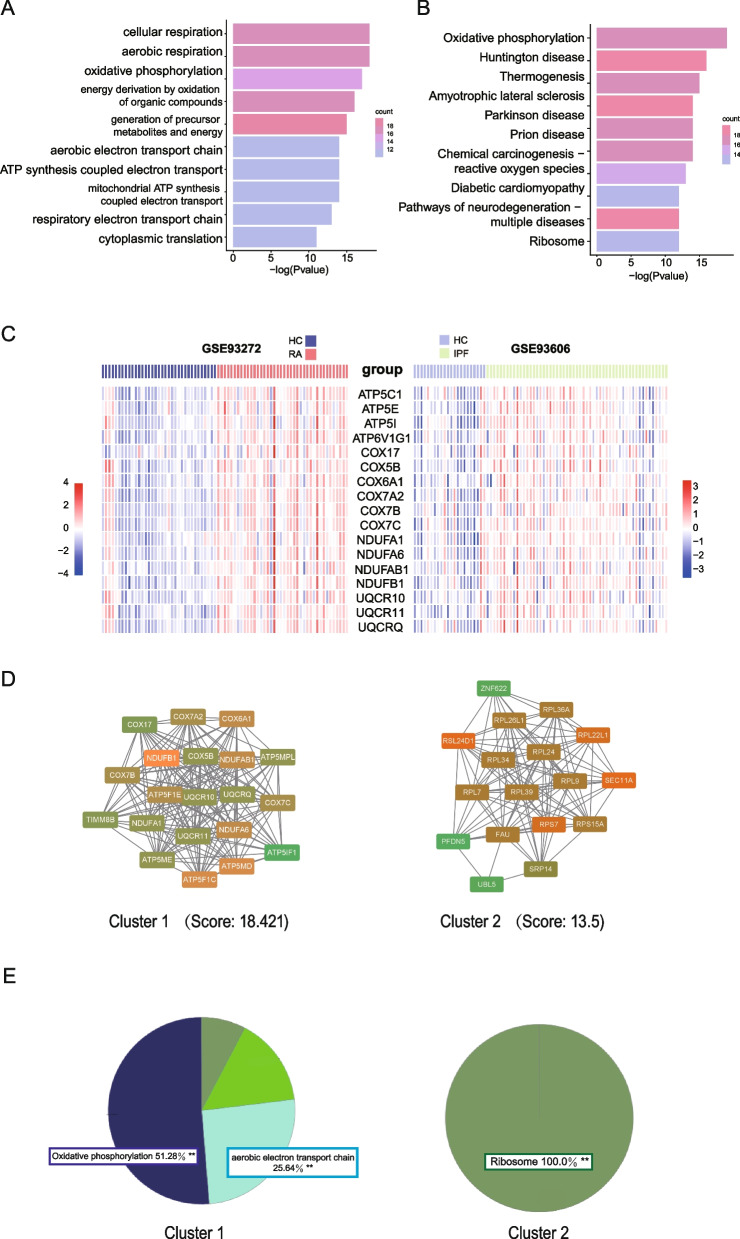
Functional enrichment and PPI analysis of RA and IPF. **A** The GO enrichment analysis results showed that cellular respiration, aerobic respiration and oxidative phosphorylation were significantly enriched in RA and IPF. **B** The KEGG enrichment analysis results showed that oxidative phosphorylation were significantly enriched in RA and IPF. **C** The heatmap showed the expression of genes related to oxidative phosphorylation in GSE93272 and GSE93606. **D** The interaction network of GO terms of the genes in cluster 1 and 2 was generated by the Cytoscape plug-in MCODE. **E** The proportion of each GO term group enriched in cluster 1 and 2. GO, gene ontology. KEGG, Kyoto Encyclopedia of Genes and Genomes. MCODE, Molecular Complex Detection

To identify shared biological characteristics between RA and IPF, we performed Gene Ontology (GO) and Kyoto Encyclopedia of Genes and Genomes (KEGG) enrichment analyses separately for the gene sets associated with each group. In the “RA yellow module related IPF gene set”, there were not enough genes available to identify significant terms. We found significant enrichment of terms related to “celluar respiration”, “aerobic respiration” and “oxidative phosphorylation” were enriched in the other gene set (Fig. 4A). Additionally, our analysis identified “oxidative phosphorylation” as the most significantly enriched pathway within the “RA blue module related IPF gene set” (Fig. 4B). These findings suggest a potential involvement of mitochondrial processes and energy metabolism, particularly oxidative phosphorylation, in the shared biological characteristics between RA and IPF. The heatmaps in Fig. 4C illustrated the expression levels of all genes associated with the "oxidative phosphorylation" both in GSE93272 and GSE93606.

We further constructed a protein–protein interaction (PPI) network using the 144 common genes identified from two diseases. Two clusters were obtained by MCODE analysis. Cluster 1 contained 20 nodes and 175 edges (score = 18.421), and Cluster 2 contained 17 nodes and 108 edges (score = 13.5) (Fig. 4D). We subjected the genes in two clusters to ClueGO analysis separately, resulting in the identification of distinct enriched terms. In one cluster, the analysis revealed enrichment of the "oxidative phosphorylation" pathway, indicating the potential involvement of this pathway, which further reinforced the potential involvement of oxidative phosphorylation in RA-UIP and IPF. In the other cluster, the analysis highlighted the enrichment of the "ribosome" term, suggesting the significance of ribosomal components and related processes within the genes in cluster 2 (Fig. 4E).

### Validation of the selected genes in a RA pulmonary RNA sequencing data set and collagen-induced arthritis (CIA) arthritis model

**Fig. 5:**
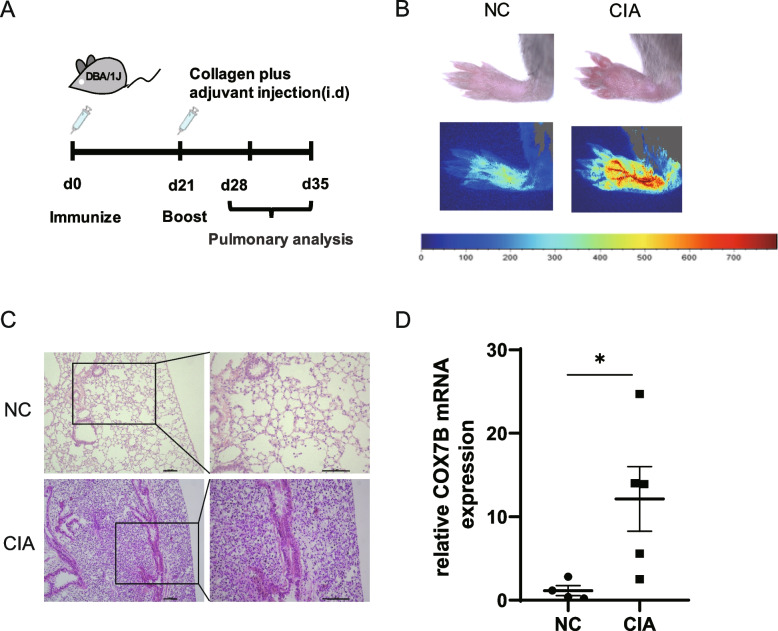
Validation of selected genes in the CIA mouse model. **A** Schematic diagram of the CIA mouse model. **B** Arthritis manifestations in CIA mice. **C** HE staining of lung tissues of CIA and normal control mice. **D** COX7B mRNA expression in lung tissue of CIA and normal control mice. Values are mean ± SD. *P < 0.05

In Fig. 5A, we identified the top 10 genes with the highest node scores in cluster 1 as hub genes, including NDUFB1, ATP5MD, ATP5F1C, NDUFA6, NDUFAB1, COX6A1, ATP5F1E, COX7A2, COX7B, COX7C. We further validated these selected genes as representative of the common characteristics in lung tissues of patients with RA-UIP and IPF-UIP in GSE199152. As depicted in Supplementary Fig. S1 (Additional file 1), the expression levels of “COX7B” and “ATP5E” displayed a similar upward trend in patients with both RA-UIP and IPF-UIP as compared to non-UIP samples. However, no significant difference was found between the expression levels of these genes in RA-UIP and non-UIP, possibly due to individual variations and small sample size of RA-UIP (*n* = 3).

To better validate our results, we immunized DBA/1 J mice with collagen and Complete Freund’s Adjuvant (CFA) (Fig. 5A) so as to induce an experimental arthritis model in mice. Acute arthritis peaked within 1–2 weeks after the second collagen immunization, as evidenced by noticeable joint swelling in the mice (Fig. 5B). Lung tissue samples were obtained for HE staining, which revealed a clear infiltration of inflammatory cells near the pleura in the lungs of mice with acute arthritis (Fig. 5C). Among the 10 hub genes identified above, only COX7B mRNA expression in the lung tissue of CIA mice was found to be significantly higher than that in the normal control (NC) group (Fig. 5D), while the expression levels of the other genes showed no significant differences.

### The unique characteristics of RA-UIP in pulmonary tissues

Although sharing the similar UIP features in the lung, different mechanisms may contribute to the ILD development in RA-UIP and IPF-UIP. Therefore, we carried out further differential gene analysis and enrichment analysis on the GSE199152 dataset to explore the unique characteristic of RA-UIP.

**Fig. 6:**
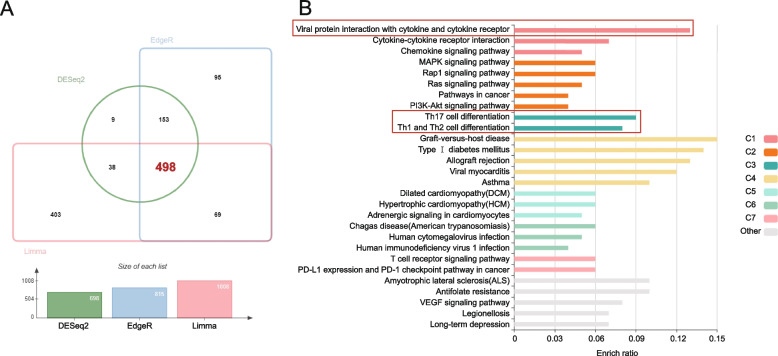
Differentially expression analysis of GSE199152. **A** DESeq2, EdgeR and Limma packages were used to filter up-regulated DEGs. **B** The up-regulated DEGs were conducted KEGG enrichment in KOBAS. The color of the bar chart denotes different groups under preset conditions of the website. KEGG, Kyoto Encyclopedia of Genes and Genomes

We used 3 conventional differential gene algorithms, including DESeq2, EdgeR, and Limma packages, to perform differential gene analyses on this dataset, and obtained 498 up-regulated genes (Fig. 6A). Further, these genes were analyzed by KEGG enrichment in KOBAS. The enriched pathways were separated into 7 clusters, interestingly, of which “Viral protein interaction with cytokine and cytokine receptor”, “Th17 cell differentiation” and “Th1 and Th2 cell differentiation” pathways closely related to RA showed high enrich ratio (0.13, 0.09 and 0.08 respectively) (Fig. 6B). These results suggest immune dysfunction may participate in the pathogenesis of RA-UIP development.

### Distribution of the immune cells in RA-UIP and IPF-UIP

**Fig. 7:**
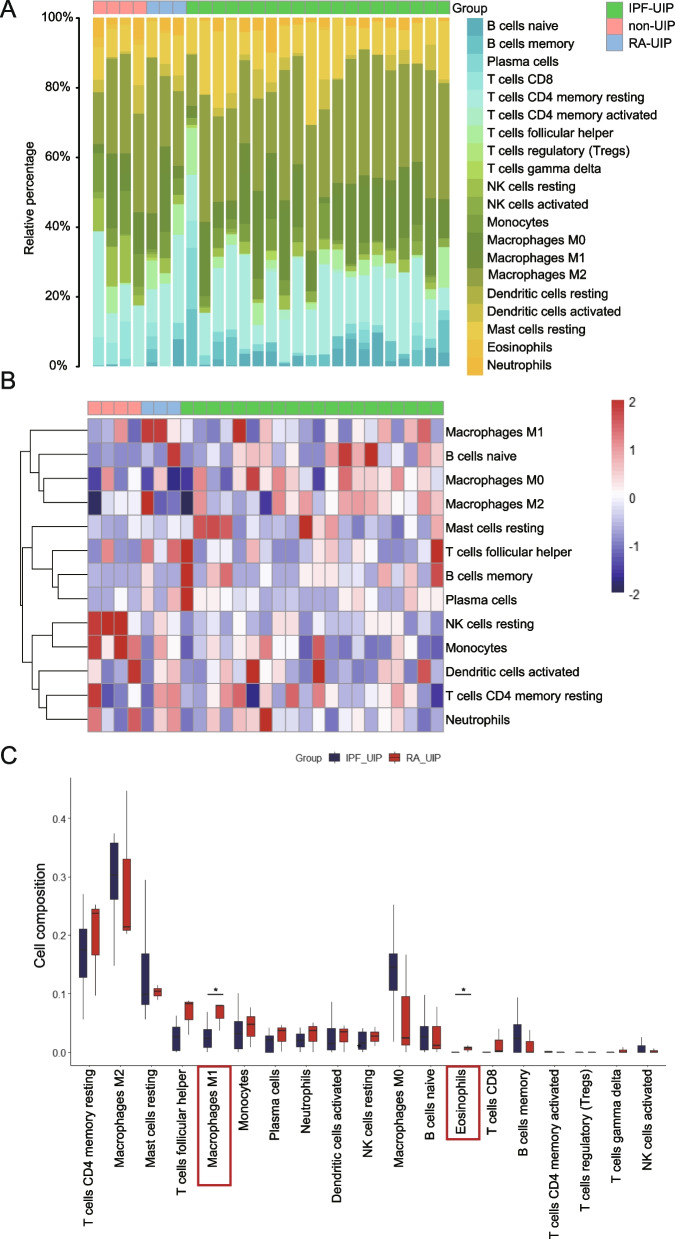
Immune infiltration analysis. **A** The stack histogram shows the relative proportion of 22 immune cell types in each sample. **B** The heatmap shows the abundance of 13 major immune cell types. Cell subsets with low expression are not present here. **C** The box plot shows the different immune cell infiltration of RA-UIP and IPF-UIP

To make a better understanding of the immunopathological mechanism underlying RA-UIP, the proportion of 22 immune cell types in lung samples from GSE199152 dataset was calculated (Fig. 7A, B). Upon comparing the levels of infiltrated immune cells in IPF-UIP and RA-UIP samples, we found that the infiltration abundance of “macrophage M1” and “Eosinophils” cells was significantly higher in RA-UIP samples, while “macrophage M0” and “macrophage M2” lower in RA-UIP samples than those in IPF-UIP (Fig. 7C). The levels of other infiltrated immune cells showed no statistical difference as visualized in the box plot.

## Discussion

Rheumatoid arthritis-associated interstitial lung disease (RA-ILD) can present as the initial manifestation of RA, with usual interstitial pneumonia (UIP) being the most common histological pattern [8]. It is indisputable that the survival rate of RA patients with UIP pattern (RA-UIP) is significantly lower than those of other pathological types [5, 14–16]. Currently, some studies have focused on the role of systemic and localized ACPAs [17, 18] and lung microbiomes [19] in RA-ILD. However, the understanding of the pathogenesis of RA-UIP is limited [20]. IPF, a fibrotic lung disease of unknown origin, is diagnosed by the presence of the UIP pattern on histopathology and/or CT imaging. The environmental, phenotypical and genetic similarities between RA-UIP and IPF suggest that they may share similar underlying pathological mechanisms.

With the rapid development of high-throughput sequencing technology, it is possible to effectively mine underlying mechanisms of RA-UIP from massive data. In recent years, WGCNA has been applied to explore the biological characteristics of humans, plants, and donkeys [21–23]. In the current study, we firstly used WGCNA method to construct a co-expression network of IPF-related RA genes in the whole blood of patients with RA and IPF based on array data from GEO datasets (GSE93272, GSE93606). A total of 4 (RA-positive related) and 5 (IPF-positive related) modules were identified in GSE93272 and GSE93606, respectively.

Next, we conducted PCA and differential expression analysis on these two datasets, and found a total of 553 overlapped DEGs. Only those intersection genes in RA and IPF modules with significant expression (144 genes) were subjected to further analysis under pre-set filtering criteria. Through the conducted enrichment analysis and PPI analysis, our study revealed that “oxidative phosphorylation” was the most relevant biological process of RA and IPF. As the RA-UIP exhibits the convergence of clinical characteristics from RA and IPF, it indicated that the disease may share the common molecular mechanisms of RA and IPF. To validate our findings, we detected the hub genes in GSE199152 (a RNA sequencing dataset includes pulmonary tissues from non-UIP, RA-UIP, and IPF-UIP patients), and found that the expression of most genes showed an upward trend in RA-UIP and IPF-UIP as compared with non-UIP. These genes are involved in oxidative phosphorylation related pathways, suggesting that the process of lung fibrosis in RA-UIP and IPF-UIP requires constant energy metabolism adaptation to meet the metabolic demands in lung fibroblast. A transcriptome sequencing for a larger size of RA-UIP pulmonary samples is needed to clear and further validate the mechanism.

In the most of previous studies, researchers have primarily focused on investigating the glycolytic phenotype. However, emerging evidence indicates that myofibroblasts in IPF lung tissue also exhibit an increase in mitochondrial respiration [24]. The increased mitochondrial respiration in fibrotic conditions provides adenosine-triphosphate (ATP), TCA carbon intermediates, and reactive oxygen species (ROS), all of which contribute to supporting and driving the fibrogenic responses observed in diseases like IPF [25, 26]. Rangarajan et al. also discussed the abnormal state of mitochondrial in aged fibroblasts of IPF [27], highlighting the potential role of mitochondrial dysfunction in the pathogenesis of the disease. On the other hand, M. Biniecka et al. had focused on the topic of cellular bioenergetics and its association with synovial inflammation in RA [28]. However, we could not identify any relevant articles discussing the alteration of oxidative phosphorylation in RA-UIP, which may due to the challenges encountered in conducting fundamental research in RA-UIP. In our study, we have taken the initiative to explore the potential involvement of energy changes and oxidative phosphorylation in the progression of RA-UIP. Further basic studies are warranted to provide stronger evidence and support for the finding.

Collagen-induced arthritis (CIA) is a well-established animal model for RA. In this model, the histological pattern of the lung tissue of CIA mice has been observed to be similar to that of patients with RA-associated UIP, characterized by the presence of inflammatory cell aggregates under the pleura [29]. Therefore, we induced CIA mice and found obvious inflammatory cell infiltration around trachea, blood vessels and near pleura of lungs. In a previous basic study, collagen deposition was observed during the later stages of the CIA model. The evaluation of collagen deposition was specifically conducted at 94 days post-modeling, utilizing the measurement of hydroxyproline content as an indicator [29]. Furthermore, we detected mRNA levels of cytochrome c oxidase subunit 7B (COX7B) significantly increased in the lung of CIA mice compared to normal mice. COX7B is a structural component of mitochondrial electron transport chain complex IV, which is an important part of energy metabolism [30]. The upregulation of COX7B, a component associated with oxidative phosphorylation, indicates an enhanced respiratory capacity and increased energy production within the lung [30]. These findings underscore the significance of energy metabolism in the pathogenesis of RA-UIP, highlighting the potential interplay between metabolic adaptations and the development of pulmonary fibrosis.

To better understand the unique characteristics of RA-UIP, we used GSE199152 for further comparative analysis to find the possible pathogenesis of RA-UIP. A total of 498 RA-UIP up-regulated genes were screened out for subsequent enrichment analysis. Functional annotation showed the impaction of adaptive immune response to the RA-UIP. “Hsa04061: Viral protein interaction with cytokine and cytokine receptor” has the most significant correlation with RA-UIP. It has been reported that cytokines, such as TNF-α, IL-6 and IL-17 play a critical role in the development of RA and RA-related extra-articular symptom. Our data imply that cytokine and cytokine receptor might contribute to inflammation and subsequent repair in lung tissue of RA. In addition, it is possible that disease-modifying anti-rheumatic drugs (DMARDs) or biological agents used in RA may increase the risk of viral infection in lung, which might participate in ILD development to some extent [31].

In addition, “hsa04659: Th17 cell differentiation; hsa04658: Th1 and Th2 cell differentiation” were also enriched in our analysis. Th1, Th2 and Th17 have previously been proved to involve in the pathogenesis of RA-ILD. CD4^+^lymphocytes increased in lung biopsy samples of RA-UIP patients [32], as well as Th17 cells participated in pulmonary fibrosis by release of IL-17A and TGF-β promoting the proliferation of fibroblasts and the formation of extracellular matrix [33]. Moreover, the CIA mouse model has demonstrated an increase in Th1, Th2, and Th17 cells within the lung tissue, as well as the deposition of auto-antibodies, such as anti-citrullinated fibrinogen antibodies, which is consistent with the observed elevation of ACPA in RA-UIP patients [34–37]. The present bioinformatic analysis, along with previous research, indicates a potential involvement of Th1, Th2, and Th17 cell subtypes in the pathogenesis of RA-UIP, and this process may have a strong association with the presence of ACPA.

In order to gain a deeper understanding of the immunopathological mechanisms underlying the development of RA-UIP, an immune infiltration analysis of RA-UIP and IPF-UIP was conducted. Our analysis revealed that the proportion of "Macrophage M1" cells was significantly higher in RA-UIP compared to IPF-UIP, whereas M0 and M2 cell subsets exhibited a lower percentage in RA-UIP without statistical significance compared to IPF-UIP. M2 macrophages secrete anti-inflammatory molecules and play a profibrotic role in IPF, as observed in bleomycin-induced IPF in rats [38–41]. This specific type of macrophage is capable of recruiting and increasing proliferation of fibroblasts, so as to promote their differentiation into myofibroblasts and facilitate the deposition of extracellular matrix (ECM) [42]. On the other hand, M1 macrophages are known to produce pro-inflammatory cytokines such as TNF-α and IL-1, which potentially were triggered by autoimmune reactions, exacerbating the development of RA-UIP. In a previous study, it was discovered that Toll-like receptor 4 (TLR4), a surface biomarker predominantly associated with M1 macrophages, was involved in the increased synthesis and phosphorylation of ECM components. This finding suggests a potential contribution of TLR4 in M1 phenotype macrophage to the development of RA-UIP [43]. However, the detailed mechanism by which TLR4 influences ECM synthesis and phosphorylation in the context of RA-UIP remains unknown.

## Conclusions

In conclusion, our findings suggest that the genesis and progression of RA-UIP may be associated with dysregulated oxidative phosphorylation, as well as aberrant immune responses involving M1 macrophages and T cells subsets.

## Methods

### GEO datasets download and processing

We use the keyword “rheumatoid arthritis” or “idiopathic pulmonary fibrosis” to search for gene expression profiling tested by microarray or high-throughput sequencing from the National Center for Biotechnology Information (NCBI) Gene Expression Omnibus (GEO). The criteria were as follows: (1) the gene expression profiling must include cases and controls, (2) the organization used for sequencing should be whole blood, and (3) the number of samples in each group should not be less than 20 to ensure the accuracy of WGCNA. Two datasets (GSE93272 and GSE93606) were included for WGCNA analysis. GSE93272 contained 40 RA patients and 35 healthy controls, and GSE93606 included 60 IPF and 20 healthy controls.

We used the keywords “rheumatoid arthritis” and “usual interstitial pneumonia” to search RA-UIP-related microarray or high-throughput sequencing datasets in the GEO database, and obtained GSE199152 including pulmonary tissues from 3 RA-UIP patients, 20 IPF-UIP patients, and 4 non-UIP patients. The dataset GSE199152 was used to verify the results of WGCNA and perform differential gene analysis. The normalization of reads count was performed to transcripts per million (TPM) and subsequently used to compare the expression levels between the groups.

For microarray data, the Series Matrix Files provided by the contributors were used to obtain sample information and expression of probes. According to the annotation file of the corresponding platform, we matched the probe with its gene symbol and obtained the average value of different probes of the same gene through the algorithm. For data from high-throughput sequencing datasets, count files provided by the contributors were obtained for subsequent analysis. The outline of the workflow is shown in Fig. 1.

### WGCNA network construction and module identification

The WGCNA approach is widely used to study global coexpression across all samples to identify clusters of highly correlated genes associated with diseases [44]. Therefore, we used WGCNA to obtain RA and IPF-related modules.

WGCNA analysis was performed with the “WGCNA” package in R software (version 4.0.3). The “goodSamplesGenes” function in the “WGCNA” R package was used to remove genes with too many missing samples or zero variance, and then the 10,000 genes exhibiting the highest mean expression values were then selected for further analyses. The “hclust” function in the “stat” R package was used to perform hierarchical cluster analysis to exclude abnormal samples, and the “pickSoftThreshold” function in the “WGCNA” R package was used to analyze the network topology, aiming to pick an appropriate soft-thresholding power β for network construction. The topological overlap matrix (TOM) was further generated using the “TOMsimilarity” function. Hierarchical clustering analyses were then conducted again to detect modules based on the TOM. Last, the “moduleEigengenes” function was used to calculate module eigengenes of modules. Therefore, the modules with a high positive correlation coefficient with the disease were focused on in our analyses.

In this study, the soft threshold β was 3 in the WGCNA analysis of GSE93272 and GSE93606. The other parameters were the following: minClusterSize = 50, cutheight = 0.2.

### Differential expression analysis

The differentially expressed genes (DEGs) between patients and healthy controls of datasets GSE93272 and GSE93606 were performed by the “Limma” package in R software [45]. The *p* value < 0.05 and |log_2_-fold change (FC)|> 0.1 were considered to be statistically significant.

The UP-DEGs between RA-UIP patients and IPF-UIP patients of dataset GSE199152 were obtained by using the “limma” package [38], “DESeq” package [46] and “EdgeR” package [47] in R, with the criteria of “*P* < 0.05 and |log_2_FC|> 1”. Jvenn, an online tool for generating Venn diagrams [48], was used to obtain the intersect UP-DEGs of 3 algorithms, which will be used for further analysis.

### Principal Component Analysis (PCA)

PCA is a mathematical algorithm used to explore high-dimensional datasets [49]. It reduces the data’s dimensionality and identifies variation patterns within the data. In this study, “FactoMineR” [50] and “factoextra” [51] packages in R software were used for analysis and visualization.

### Protein–Protein Interaction (PPI) network construction

The PPI network of DEGs was predicted using the online tool STRING [52] with a combined score > 0.7. Cytoscape software (v3.9.1) was used to achieve better visualization. Molecular Complex Detection (MCODE) algorithm was used to identify highly interconnected subclusters within the input genes. Gene ontology annotations were performed using the ClueGO 2.5.9 app [53]. ClueGO is a plug-in of Cytoscape software, which could ensure an up-to-date functional analysis and visualize functionally grouped terms in the form of networks.

The list of RA-related IPF genes was selected to input to MCODE and ClueGO, and parameters were applied with default values. Gene Ontology (GO) terms (biological process, cellular component and molecular function) were selected as the annotation sources.

### Functional enrichment analysis

Kyoto Encyclopedia of Genes and Genomes (KEGG) analysis is widely used to understand biological mechanisms and functions [54, 55]. The UP-DEGs of GSE199152 have conducted a KEGG analysis on KOBAS [56]. The criteria are *P* < 0.05. Enriched terms are visualized in the form of a histogram. The color of the bar represents different clusters.

### Evaluation of immune cell infiltration

To evaluate the proportion of infiltrating immune cell subsets in RA-UIP, gene expression matrix file of GSE199152 were uploaded to the CIBERSORTx [57] and LM22 (22 immune cell types) gene signatures were used to annotate them. Then, we compared the distribution of the 22 immune cells between RA-UIP and IPF-UIP groups. The “ggplot2” package in R software was used for visualization.

### Collagen-Induced Arthritis (CIA) in DBA/1 J mice

The induction of CIA was described previously [58]. Briefly, 6 week-old DBA/1 J male mice (Shanghai SLAC Laboratory Animal Co.,Ltd) were immunized at the base of the tail with 200 µg bovine CII (Chondrex, USA) emulsified with CFA (Chondrex, USA), and boosted by immunization on day 21 in the same manner. The animal experiments were performed in accordance with the guidelines approved by Institutional Animal Care and Use Committee of Nanjing Medical University. The arthritis of mice was optically evaluated by using laser speckle imaging equipment (moorFLPI V5.0, Moor Instruments Ltd, UK).

### Hematoxylin and Eosin (HE) staining

For histological assessment, lungs of the mice were obtained after anesthesia by 1% pentobarbital sodium. Take the lung tissue to flush the lung blood away and then fixed overnight in 4% paraformaldehyde. The material was then embedded with paraffin and cut into 4–6 µm for HE staining. Microscopic images were captured with a microscope (Nikon Eclipse 50i, Japan).

### RNA extraction and Real-Time Quantitative PCR (RT-PCR)

Total RNA was extracted using TRIzol reagent (Invitrogen, Carlsbad, CA, USA) and cDNA was synthesized by the RNA PCR Core Kit (Applied Biosystems, Branchburg, NJ, USA). RT-PCR was performed using an ABI Prism 7900 Sequence Detection System. Relative expression was calculated with normalization to GAPDH values by using the 2^−ΔΔCt^ method. The primer sequences used for the target genes were as follows: mouse COX7B forward, 5’-TTGCCCTTAGCCAAAAACGC-3’ and reverse, 5’-TCATGGAAACTAGGTGCCCTC-3’; GAPDH forward, 5’-GTCTCCTCTGACTTCAACAGCG-3’ and reverse 5’-ACCACCCTGTTGCTGTAGCCAA-3’. All experiment data are expressed as means ± standard deviation (SD), and statistical analysis was performed by a 2-tailed unpaired Student *t* test.* P* less than 0.05 was considered statistically significant.

### Supplementary Information

**Additional file 1: Figure S1.** Verification of the expression of hub genes in pulmonary tissue. The relative expression (R.E.) of 10 selected gene in pulmonary tissue of GSE199152. (A) COX6A1; (B) COX7A2; (C) COX7C; (D) COX7B; (E) NDUFAB1; (F) NDUFB1; (G) NDUFA6; (H) USMG5; (I) ATP5C1; (J) ATP5E. The gene expression levels were normalized from the raw read count data using TPM normalization.

**Additional file 2: Table S1.** The intersection of genes in RA modules and IPF modules.

**Additional file 3: Table S2.** “RA yellow module related IPF gene set” and “RA blue module related IPF gene set”.

## Data Availability

The datasets used and/or analysed during the current study are available from the corresponding author on reasonable request.

## Abbreviations

RA: Rheumatoid arthritis
RA-UIP: Rheumatoid arthritis-related usual interstitial pneumonia
GEO: Gene Expression Omnibus
IPF: Idiopathic pulmonary fibrosis
WGCNA: Weighted gene co-expression network analysis
DEG: Differentially expressed gene
ILD: Interstitial lung disease
UIP: Usual interstitial pneumonia
NSIP: Non-specific interstitial pneumonia
ACPA: Anti-citrullinated protein antibodies
HLFs: Human lung fibroblasts
SNP: Single nucleotide polymorphism
TPM: Transcripts per million
CIA: Collagen-induced arthritis
PPI: Protein-protein interaction
NCBI: National Center for Biotechnology Information
GO: Gene Ontology
KEGG: Kyoto Encyclopedia of Genes and Genomes
TOM: Topological overlap matrix
HRCT: High resolution CT
SD: Standard deviation
ECM: Extracellular matrix
TLR4: Toll-like receptor 4
