# Supplementary material for: Integrating bioinformatic resources to identify characteristics of rheumatoid arthritis-related usual interstitial pneumonia
Source: BMC Genomics. 2023 Aug 10;24:450. doi: 10.1186/s12864-023-09548-2 (PMC10413595; doi:10.1186/s12864-023-09548-2)
Supplement: Supplementary file 1 — Additional file 1: Figure S1. Verification of the expression of hub genes in pulmonary tissue. The relative expression (R.E.) of 10 selected gene in pulmonary tissue of GSE199152. (A) COX6A1; (B) COX7A2; (C) COX7C; (D) COX7B; (E) NDUFAB1; (F) NDUFB1; (G) NDUFA6; (H) USMG5; (I) ATP5C1; (J) ATP5E. The gene expression levels were normalized from the raw read count data using TPM normalization. [file 12864_2023_9548_MOESM1_ESM.pdf]

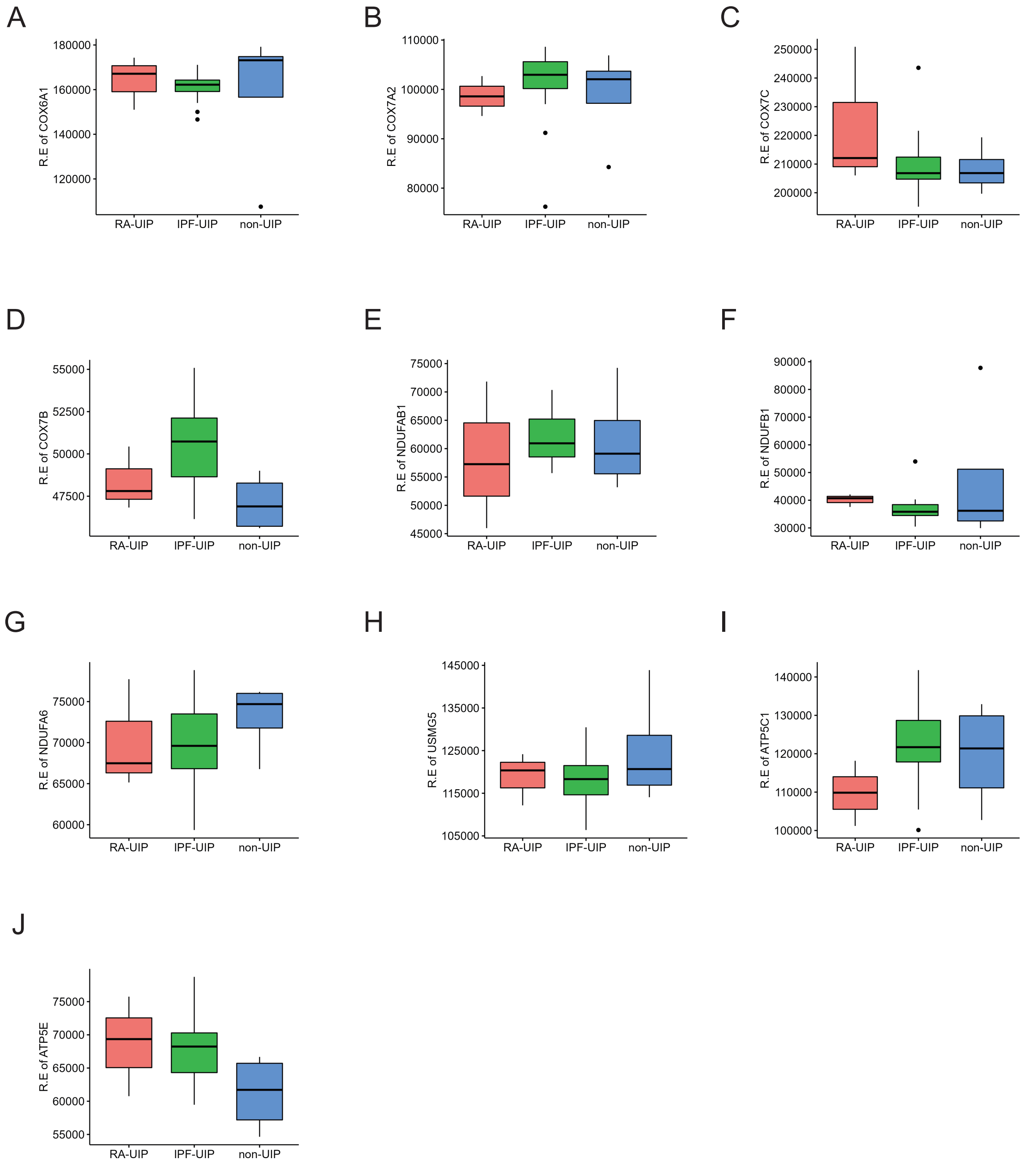

Figure S1

Verification of the expression of hub genes in pulmonary tissue. The relative expression (R.E.) of 10 selected gene in pulmonary tissue of GSE199152. (A) COX6A1; (B) COX7A2; (C) COX7C; (D) COX7B; (E) NDUFAB1; (F) NDUFB1; (G) NDUFA6; (H) USMG5; (I) ATP5C1; (J) ATP5E. The gene expression levels were normalized from the raw read count data using TPM normalization.
